# Relationship Between Child Care Centers’ Compliance With Physical Activity Regulations and Children’s Physical Activity, New York City, 2010

**DOI:** 10.5888/pcd11.130432

**Published:** 2014-10-16

**Authors:** Robert L. Stephens, Ye Xu, Catherine A. Lesesne, Lillian Dunn, Jakub Kakietek, Jan Jernigan, Laura Kettel Khan

**Affiliations:** Author Affiliations: Ye Xu, Catherine A. Lesesne, Jakub Kakietek, ICF International, Atlanta, Georgia; Lillian Dunn, New York City Department of Health and Mental Hygiene, New York, New York; Jan Jernigan, Laura Kettel Khan, Centers for Disease Control and Prevention, Atlanta, Georgia.

## Abstract

**Introduction:**

Physical activity may protect against overweight and obesity among preschoolers, and the policies and characteristics of group child care centers influence the physical activity levels of children who attend them. We examined whether children in New York City group child care centers that are compliant with the city’s regulations on child physical activity engage in more activity than children in centers who do not comply.

**Methods:**

A sample of 1,352 children (mean age, 3.39 years) served by 110 group child care centers in low-income neighborhoods participated. Children’s anthropometric data were collected and accelerometers were used to measure duration and intensity of physical activity. Multilevel generalized linear regression modeling techniques were used to assess the effect of center- and child-level factors on child-level physical activity.

**Results:**

Centers’ compliance with the regulation of obtaining at least 60 minutes of total physical activity per day was positively associated with children’s levels of moderate to vigorous physical activity (MVPA); compliance with the regulation of obtaining at least 30 minutes of structured activity was not associated with increased levels of MVPA. Children in centers with a dedicated outdoor play space available also spent more time in MVPA. Boys spent more time in MVPA than girls, and non-Hispanic black children spent more time in MVPA than Hispanic children.

**Conclusion:**

To increase children’s level of MVPA in child care, both time and type of activity should be considered. Further examination of the role of play space availability and its effect on opportunities for engaging in physical activity is needed.

## Introduction

As a result of rising childhood obesity rates, public health practitioners have examined the effects of environmental and policy change interventions to promote childhood physical activity (PA) (1–5). Previous findings indicate that preschool-aged children engage in low levels of vigorous activity and spend large amounts of time inactive (3,6). Evidence from longitudinal and cross-sectional studies suggests that PA protects against overweight and obesity among preschoolers and that policies and characteristics of group child care centers influence PA levels of children who attend them (7–10). Child care policies provide a promising strategy to address children’s PA and potential obesity (11–13).

In 2007, New York City’s (NYC’s) Department of Health and Mental Hygiene (DOHMH) implemented modified regulations governing group child care centers, establishing minimum standards for PA, beverage consumption, and television viewing for children (14). The PA-related regulations require centers to schedule at least 60 minutes of total PA per day and at least 30 minutes of structured PA per day for students in full-day classrooms. NYC DOHMH also provided PA trainings and associated technical assistance (ie, Sports, Play and Active Recreation for Kids [SPARK] and Eat Well Play Hard Training of Teachers [TOTs] curricula) (15,16). This article addresses how compliance with requirements to offer at least 30 minutes of structured PA and at least 60 minutes of total PA each day is associated with children’s moderate to vigorous PA (MVPA).

## Methods

The evaluation used a multicomponent design. A center evaluation component assessed center-level compliance with regulations and organizational characteristics associated with compliance. A classroom evaluation component assessed classroom-level compliance with regulations during a 2-day period and child-level PA using accelerometers. In this issue of *Preventing Chronic Disease* (*PCD*), Breck et al present a full description of the methods (17). Institutional review boards at ICF International and NYC DOHMH approved the study protocol.

### Center selection

From among the 1,654 early child care and education centers licensed by NYC DOHMH Bureau of Child Care, a random sample of 176 eligible centers agreed to participate in the center evaluation component (fall 2009). We limited the sample to centers serving low-income communities, defined by census tracts with 40% or more of families with incomes at 200% of the federal poverty line or below and stratified by location in District Public Health Office (DPHO) catchment areas. A sample of 110 of the 176 centers participated in the classroom evaluation component (spring 2010). These 110 centers represented 63% of the invited random, stratified sample. We randomly selected 1 classroom of preschoolers per participating center for 2 days of observation.

As part of the classroom component, data collectors documented variables related to classroom compliance through observation (eg, beverages teachers served, structured and unstructured PA offered, screen time offered). Although we originally did not propose to compare or contrast results of the center and classroom methods for assessing compliance, the resulting data allowed us an opportunity to examine whether and how strongly these different compliance measures related to our outcomes of interest. Also in this issue of *PCD*, Lessard et al discuss the definitions of compliance and each element’s data source (18). Briefly, the center component assessed compliance at the center level using staff report (ie, teacher or director reported that children spent at least 30 total minutes per day in structured PA and a combined total of at least 60 total minutes per day in structured and unstructured PA). The classroom component assessed compliance with the regulations using direct observation of PA offerings in selected classrooms (ie, data collectors observed all structured and unstructured PA events offered to determine if amounts totaled at least 30 minutes of structured PA and at least 60 minutes for all structured and unstructured PA).

### Participants

Eligible participants included 1,465 children from the 110 participating centers. The resulting sample included 1,352 children who participated in accelerometry data collection and had complete data. Our analysis excluded children aged younger than 2 years, 10 months or older than 5 years, 11 months or those with missing date-of-birth information (n = 38). The analysis also excluded children who experienced malfunctions of accelerometer equipment (n = 72) or an error in assignment of the accelerometers worn during the 2-day observation period (n = 3). Approximately one-fifth of children (21.7%) wore accelerometers for only 1 day; they were included in the sample. A comparison of children included with those excluded showed that the only significant difference was that excluded children were slightly younger (included: mean, 3.39 y; excluded: mean, 3.27 y).

### Data collection and analysis methods

Data on children’s heights and weights were collected to assess body mass index (BMI), and accelerometers measured duration and intensity of PA. Children wore accelerometers only while in the child care center. Before arrival on the first day of data collection, data collectors recalibrated accelerometers to record a 15-second epoch. Because of an error in recalibration, 140 children wore accelerometers set to a 1-minute epoch rather than a 15-second epoch. These children were included in the sample, and the analyses were adjusted for their inclusion.

On the first day of observation, data collectors placed an accelerometer (ActiGraph GT3X) on each participating child. We calculated accelerometer wear times using the recorded times at which belts were put on and taken off children, and adjusted for likely nonwear time (defined below). Procedures were repeated the second day with the same accelerometer used both days (except for those children who wore accelerometers only 1 day). The accelerometers were to be worn on both days of classroom data collection. Although 1 to 2 days of accelerometry data are below the number of days recommended to provide reliable estimates of PA, the procedure needed to be brief, as it was administered with 3-year-olds in actual day care centers during normal operation. 

Accelerometry data were processed using an Excel macro program developed by Stewart Trost and used with his permission (S. Trost, personal communication). Cut points developed for preschool-aged children by Pate and colleagues (19) were used to determine how much time each child engaged in different levels of PA. Because children wore accelerometers for varying lengths of time, we calculated minutes per hour of wear time to standardize data across children. For children who wore accelerometers both days, we calculated minutes of wear time per hour for each day separately and then averaged across both days.

According to the Institute of Medicine’s Early Childhood Obesity Prevention Policies, toddlers and preschool-aged children should be provided opportunities for 15 minutes of light, moderate, and vigorous PA per hour while in care (20). To be consistent with Institute of Medicine recommendations, we analyzed MVPA among the children participating in this study. The calculated PA levels included sedentary (eg, sitting), light (eg, slow walking), moderate (eg, fast walking, skipping), and vigorous (eg, running). Accelerometry data were classified as follows: sedentary PA (<100 average counts/minute), light PA (≥100 and <1,680 average counts/minute), moderate PA (≥1,680 and <3,368 average counts/minute), vigorous PA (≥3,368 average counts/minute). Thus, MVPA included periods with total average counts greater than or equal to 1,680. Periods with consecutive zero counts lasting 60 minutes or longer were defined as nonwear time and were excluded from total wear time.

### Calculation of compliance with PA regulations

Centers’ compliance scores were calculated using procedures described in this issue of *PCD* by Lessard et al (18). Categories of compliance were as follows: 1) compliant with center and classroom components, 2) compliant with center component but not classroom component, 3) compliant with classroom component but not center component, and 4) compliant with neither component. In these analyses, we included 2 binary indicators: 1) consistently compliant with the regulation of at least 30 minutes of structured PA per day in both center and classroom components, and 2) consistently compliant with the regulation of at least 60 minutes of total PA per day in both center and classroom components.

### Variables and analysis approach

To account for clustering of children within centers, we used 2 hierarchical linear models that consisted of 2-level generalized linear regressions assessing effects of center- and child-level factors on child-level PA. Covariates from both center and classroom components were examined for multicollinearity. Only the indicators of compliance with the regulations for structured PA and total PA exhibited evidence of collinearity. We then examined bivariate relationships among variables theorized to have effects on PA. Variables with significant bivariate relationships were included in the final models. Given their collinearity, the 2 compliance variables were included in separate models. All other covariates were included in both models. All analyses were conducted using Stata version 11 (StataCorp LP).

All continuous covariates were grand-mean centered to facilitate interpretation of intercepts as mean minutes per hour spent in MVPA when covariates were held at referent or grand-mean values. Center-level covariates included Child and Adult Care Food Program (CACFP), Head Start, and NYC DPHO status; mean classroom size; operating hours; student:teacher ratio; teacher turnover; and indicators of staff PA training (ie, SPARK and TOT) and play space availability (ie, dedicated outdoor, shared outdoor, or indoor). Child-level covariates included age, sex, race/ethnicity, and BMI. As noted, because of a recalibration error, 140 accelerometers had a 1-minute epoch setting. The potential effect of this difference was accounted for by including an indicator of epoch setting (1 = 1-minute, 0 = 15-second) in the models.

## Results

### PA levels of children

Boys spent significantly more time in MVPA than girls (Table 1). Levels of MVPA did not differ significantly for other child-level characteristics.

**Table 1:** Mean Minutes Per Hour for Child-Level Moderate to Vigorous Physical Activity (MVPA) Among Children in New York City Group Child Care Centers, 2010

Characteristic	MVPA, Mean (Standard Deviation)
**Age, y (n = 1,352)**	
2	5.20 (2.08)
3	6.14 (3.52)
4	6.26 (3.57)
5	6.22 (3.33)
**Sex (n = 1,351)**	
Male	6.67 (3.82)
Female	5.70 (3.12)^a^
**Body mass index (n = 1,352)**	
Underweight	6.00 (3.21)
Normal	6.10 (3.51)
Overweight	6.08 (3.31)
Obese^b^	6.65 (3.67)

a *P* < .001.

b Based on guidelines of the National Heart, Lung, and Blood Institute (21).

The number of centers considered compliant varied by evaluation component. For the regulation of at least 30 minutes of structured PA, 84 of 107 centers with complete data (78.5%) complied using the center component, and 32 of 107 centers with complete data (29.9%) complied using the classroom component. For the regulation of at least 60 minutes of total PA, 95 of 109 centers with complete data (87.2%) complied using the center component, and 28 of 109 centers with complete data (25.7%) complied using the classroom component. Across components, 23 of 107 centers with complete data (21.5%) consistently complied (ie, complied with using both evaluation components) with the structured PA regulation, and 33 of 109 centers with complete data (30.3%) consistently complied with the total PA regulation.

Children’s levels of PA varied by centers’ compliance with PA regulations (Table 2). Children in centers consistently compliant with both center and classroom components spent more time in MVPA than children in centers not consistently compliant. Centers did not differ in amount of time children spent in MVPA by compliance with the regulation for at least 30 minutes of structured PA.

**Table 2:** Children’s Mean Minutes Per Hour of Moderate to Vigorous Physical Activity (MVPA), by Child Care Center Compliance, New York City, 2010

Structured Physical Activity Regulation, Duration of MVPA	Not Compliant With Center Component or Classroom Component	Compliant With Center Component	Compliant With Classroom Component	Compliant With Center and Classroom Components
	Mean (Standard Deviation)			
30 Minutes	n = 162	n = 729	n = 124	n = 301
	6.29 (3.76)	6.17 (3.32)	5.64 (3.76)	6.42 (3.68)
60 Minutes **a**	n = 128	n = 744	n = 65	n = 403
	5.22 (3.07)	6.02 (3.28)	6.25 (3.84)	6.72 (3.89)

a *P* < .001.

### Relationships among compliance, covariates, and PA levels

Table 3 presents results for compliance with the regulation for 30 minutes of structured PA per day; Table 4 presents results for compliance with the regulation for 60 minutes of total PA per day. For the first model, the intra-class correlation (ICC) was 0.161; for the second model, the ICC was 0.169. These findings indicate that in both models, more than 15% of the total variation in MVPA was attributable to the center level (ie, variation among centers in children’s MVPA).

**Table 3:** Minutes Per Hour of MVPA Among Children in Group Child Care Centers, by Compliance With 30 Minutes of Structured PA, New York City, 2010

Variable	β	SE
**Level 2 — Center-Level Variables (n = 103)**		
**Consistently compliant with 30 minutes of structured activity **	−0.089	0.39
**Center characteristics**		
CACFP^a^	−0.51	0.54
Head Start^b^	0.25	0.40
DPHO^c^ area/technical assistance	0.51	0.32
Average classroom size	0.05	0.05
Daily operating hours (total)	−0.10	0.19
Student:teacher ratio	−0.06	0.06
Teacher turnover rate	0.91	1.30
**Training**		
Center participated in SPARK^d^	−0.11	0.73
No. of physical activity trainings other than SPARK	−0.62	0.35
No. of center staff trained in 1st SPARK	0.00	0.02
No. of center staff trained in 2nd SPARK	−0.01	0.05
No. of center staff trained in TOT^e^	0.06	0.08
No. of classroom staff trained in SPARK	0.24	0.16
No. of classroom staff trained in TOT	0.04	0.19
**Infrastructure**		
Indoor play space	0.08	0.36
Outdoor play space	0.92^f^	0.36
Shared outdoor play space	0.08	0.42
**Level 1 — Child-Level Variables (n = 1,278)**		
**Age**	0.09	0.18
**Male**	1.05^g^	0.19
**Race** h		
Non-Hispanic black	0.61^f^	0.24
Other (including white)	−0.27	0.40
**Body mass index (*z* score)**	0.12	0.08
**1-min epoch**	−0.20	0.54
**Constant**	4.50^g^	0.94

Abbreviation: MVPA, moderate to vigorous physical activity.

a The Child and Adult Care Food Program (CACFP) is a program of the US Department of Agriculture that administers federal grants to state health departments to provide nutritious meals and snacks to low-income people.

b Head Start is a comprehensive developmental program for preschool-aged children and their families who earn a household income below the federal income poverty threshold administered by the Administration for Children and Families in the US Department of Health and Human Services.

c District Public Health Offices (DHPO) is a program of the New York City Department of Health and Mental Hygiene (NYC DOHMH), which targets resources to high-need neighborhoods in the South Bronx, East and Central Harlem, and North and Central Brooklyn. These centers all received 2 individualized on-site technical assistance sessions.

d Sports, Play and Active Recreation for Kids (SPARK) is a physical activity training program that NYC DOHMH provides free of charge to licensed child care centers.

e Eat Well Play Hard Training of Teachers (TOT) is a NYC DOHMH technical assistance program that provides child care center staff the skills necessary to lead Eat Well Play Hard nutrition and physical activity curriculum in their classrooms.

f *P* = .01

g *P* < .001.

h Hispanic ethnicity served as the reference category.

**Table 4:** Minutes Per Hour of MVPA Among Children in Group Child Care Centers, by Compliance With 60 Minutes of Total PA, New York City, 2010

Variable	β	SE
**Level 2 — Center-Level Variables (n = 103)**		
**Consistently compliant with 60 minutes of total activity**	0.94^a^	0.33
**Center characteristics**		
CACFP^b^	−0.93	0.53
Head Start^c^	0.43	0.39
DPHO^d^ area/technical assistance	0.54	0.32
Average classroom size	0.08	0.05
Daily operating hours (total)	−0.12	0.19
Student:teacher ratio	−0.08	0.06
Teacher turnover rate	1.27	1.27
**Training**		
Center participated in SPARK^e^	−0.43	0.68
No. of physical activity trainings other than SPARK	−0.62	0.34
No. of center staff trained in 1st SPARK	0.01	0.02
No. of center staff trained in 2nd SPARK	0	0.05
No. of center staff trained in TOT^f^	0.08	0.08
No. of classroom staff trained in SPARK	0.25	0.16
No. of classroom staff trained in TOT	0.13	0.18
**Infrastructure**		
Indoor play space	−0.04	0.35
Outdoor play space	0.73^g^	0.36
Shared outdoor play space	0.15	0.40
**Level 1 — Child-Level Covariates (n = 1,278)**		
**Age**	0.08	0.18
**Male**	1.02^h^	0.18
**Race^i^**		
Non-Hispanic black	0.60^j^	0.24
Other (including white)	−0.15	0.39
**Body mass index (*z* score)**	0.12	0.07
**1-min epoch**	−0.08	0.51
**Constant**	4.83^h^	0.90

Abbreviations: MVPA, moderate to vigorous physical activity; SE, standard error.

a *P* = .005.

b The Child and Adult Care Food Program (CACFP) is a program of the US Department of Agriculture that administers federal grants to state health departments to provide nutritious meals and snacks to low-income people.

c Head Start is a comprehensive developmental program for preschool-aged children and their families who earn a household income below the federal income poverty threshold administered by the Administration for Children and Families in the US Department of Health and Human Services.

d District Public Health Offices (DHPO) is a program of the New York City Department of Health and Mental Hygiene (NYC DOHMH) which targets resources to high-need neighborhoods in the South Bronx, East and Central Harlem, and North and Central Brooklyn. These centers all received 2 individualized on-site technical assistance sessions.

e Sports, Play and Active Recreation for Kids (SPARK) is a physical activity training program that NYC DOHMH provides free of charge to licensed child care centers.

f Eat Well Play Hard Training of Teachers (TOT) is a NYC DOHMH technical assistance program that provides child care center staff the skills necessary to lead Eat Well Play Hard nutrition and physical activity curriculum in their classrooms.

g *P* = .04.

h *P* < .001.

i Hispanic ethnicity served as the reference category.

j *P* = .01.

On average, children spent between 4 and 5 minutes per hour in MVPA. At the center level, amount of time spent in MVPA was not associated with consistent compliance with the regulation for 30 minutes of structured PA per day (Table 3). However, amount of time spent in MVPA was significantly associated with consistent compliance with the regulation for 60 minutes of total PA per day. Children attending centers consistently compliant with this regulation spent nearly 1 minute longer per hour in MVPA than those attending centers that were not consistently compliant with this regulation (Table 4).

Among other center-level covariates, results for both models were similar. Only availability of dedicated outdoor play space was significantly associated with MVPA. Children attending centers with dedicated outdoor play spaces spent nearly 1 minute more per hour in MVPA than those attending centers that did not have dedicated outdoor play spaces (Tables 3 and 4).

At the child level, results for the models were similar. Male children spent more time in MVPA than girls (reference category), and children who were non-Hispanic black spent more time in MVPA than those who were Hispanic (reference category). A 1-minute accelerometer epoch had no significant effect on amount of time spent in MVPA, supporting inclusion of these data in the models (Tables 3 and 4). (Results of models for light PA and sedentary PA [not presented] are available upon request from the corresponding author.)

## Discussion

We examined how compliance with NYC’s regulations on PA related to amount of time children in group child care centers engage in MVPA. Although previous studies have analyzed state policies for PA in child care centers (11–13), few have examined the effect of compliance with the policies on children’s PA. In our study, children’s levels of MVPA were associated with implementation of regulations requiring at least 60 minutes of total PA per day in group child care centers. However, compliance with the regulation for at least 30 minutes of structured PA per day was not associated with amount of time spent in MVPA.

The findings regarding compliance suggest that regulations for 60 minutes of total PA are related to increased time spent in MVPA among this age group in child care settings, consistent with previous findings indicating that supportive environments promote MVPA (9). Centers varied in the proportions of structured and unstructured PA comprising the 60 minutes of total PA offered. Anecdotally, data collectors observing PA offerings noted that structured PA activities often involved children engaging in less movement (eg, moving or jumping in place) than did unstructured PA activities (eg, running in an outdoor play space). This finding is consistent with several previous studies that found unstructured play to be associated with more MVPA (3,10,22,23). To be compliant with the regulation for 60 minutes of total PA, staff in group child care centers who want to engage children in more MVPA per day may find it beneficial to offer more time in unstructured PA. This offering would be in addition to 30 minutes of structured PA and would be similar to Let’s Move! Child Care’s recommendation that centers should offer 60 to 120 minutes of PA per day (23). Unlike findings from previous studies (9), we found no evidence that providing teachers with trainings on implementation of quality PA opportunities in the classroom was associated with time spent in MVPA. That compliance with regulations for amount of time offered for structured PA was not significantly associated with MVPA does not necessarily imply that policies should promote only unstructured PA, but policy makers should consider these findings in determining targeted levels of PA and types of activities offered to promote PA.

Children attending centers with a dedicated outdoor play space spent significantly more time in MVPA, suggesting that time spent in outdoor play may promote MVPA. More research is needed to understand the pathways. Lessard et al report in this issue of *PCD* that centers with a dedicated outdoor play space were more likely to comply with implementing the center component (18). The potential importance of a center’s access to dedicated outdoor play space for complying with regulations and for promoting children’s MVPA is noteworthy. Centers should consider offering outdoor PA opportunities as well as types of play equipment and play space environment when implementing PA policy (9,10,13).

### Limitations

Because the evaluation began after NYC child care regulations were implemented, it was not feasible to conduct a study that tested causal hypotheses or to conduct pre–post analyses. The cross-sectional study design used multiple methods of data collection, however, and used multivariate, multilevel statistical models to strengthen our ability to isolate center-level contribution of regulation compliance to child-level outcomes while controlling for relevant covariates.

Study methods relied on both observational data collection and self-report, each of which has potential biases (eg, for observation: inaccurate or inconsistent classification, potential variation in activities/outcomes outside the period of observation; for self-report: inaccurate recall, social desirability). Training of data collectors and piloting of data collection procedures were used to refine the data collectors’ skills and comfort to reliably document observational data; still, the possibility existed for imprecise, inaccurate, or incomplete observations and inaccurate or more socially desirable survey responses.

The issue of recalibration errors for accelerometers of a small number of children was another limitation. To account for this, statistical analyses included epoch setting as a covariate. Results suggested the error had no significant effect on our findings. Additionally, the use of a 1- to 2-day period to collect accelerometry data was a limitation, because this time frame is below the number of days recommended to provide reliable estimates of physical activity. Estimates of MVPA therefore may be less reliable in this study than in others that used a longer data collection period. However, although we used a brief observation period, we contend this is acceptable given the limitations of the setting of the research and age of participants.

Finally, although efforts were made to recruit all child care centers that met study inclusion criteria, some centers declined participation. Centers that declined participation in the classroom component were less likely to have been compliant in the center component. Therefore, the findings may not be generalizable to all NYC child care centers. Given our focus on recruiting centers serving low-income areas, our findings are more likely generalizable to such centers and the children they serve.

### Implications

Our findings have implications for NYC DOHMH regarding its regulations and for other jurisdictions contemplating similar PA standards. Policy makers may consider that similar centers also may struggle to comply with regulation targets for time in PA.

Compliance with NYC’s PA regulations had mixed associations with children’s PA. Compliance with the structured PA regulation was not associated with increased MVPA, while compliance with the total PA regulation (structured and unstructured) was associated with increased MVPA. Policy makers may consider recommendations for both unstructured and structured PA to promote increases in children’s MVPA while in child care. Environmental infrastructure factors were associated with higher levels of physical activity; presence of a dedicated outdoor play space was strongly associated with an increase in time spent in MVPA. As policy makers contemplate the levels of PA deemed appropriate for children in group child care, they may consider the kinds of PA mandated and the influence of certain environmental factors on PA levels. Given that presence of shared and indoor play spaces was not associated with increased amounts of MVPA while presence of dedicated outdoor play spaces was, lack of outdoor play spaces may create potential barriers to engaging in PA. Further examination of the role of play space availability in promoting PA in child care centers would inform the nuances of the relationship. Although we provide cross-sectional evidence of the association of regulations and child-level PA behaviors, longitudinal research is needed to establish causal connections of regulations for group child care centers to children’s health outcomes.
